# Evaluation of Cerebral Cortex Function in Clients with Bipolar Mood Disorder I (BMD I) Compared With BMD II Using QEEG Analysis

**Published:** 2015-04

**Authors:** Ali Khaleghi, Ali Sheikhani, Mohammad Reza Mohammadi, Ali Moti Nasrabadi

**Affiliations:** 1 Department of Biomedical Engineering, Science and Research Branch, Islamic Azad University, Tehran, Iran; 2 Psychiatry and Psychology Research Center, Roozbeh hospital, Tehran University of Medical Sciences, Tehran, Iran; 3 Biomedical Engineering Department, Shahed University, Tehran, Iran

**Keywords:** *Bipolar Mood Disorder I*, *Bipolar Mood Disorder II*, *Quantitative Electroencephalography*, *Entropy*

## Abstract

**Objective:** Early diagnosis of type I and type II bipolar mood disorder is very challenging particularly in adolescence. Hence, we aimed to investigate the cerebral cortex function in these patients, using quantitative electroencephalography analysis to obtain significant differences between them.

**Methods:** Thirty- eight adolescents (18 patients with bipolar disorder I and 20 with BMD II) participated in this study. We recorded the electroencephalogram signals based on 10-20 international system by 21 electrodes in eyes open and eyes closed condition resting conditions. Forty seconds segments were selected from each recorded signals with minimal noise and artifacts. Periodogram Welch was used to estimate power spectrum density from each segment. Analysis was performed in five frequency bands (delta, theta, alpha, beta and gamma), and we assessed power, mean, entropy, variance and skewness of the spectrums, as well as mean of the thresholded spectrum and thresholded spectrogram. We only used focal montage for comparison. Eventually, data were analyzed by independent Mann-Whitney test and independent t test.

**Results:** We observed significant differences in some brain regions and in all frequency bands. There were significant differences in prefrontal lobe, central lobe, left parietal lobe, occipital lobe and temporal lobe between BMD I and BMD II (P < 0.05). In patients with BMD I, spectral entropy was compared to patients with BMD II. The most significant difference was observed in the gamma frequency band. Also, the power and entropy of delta frequency band was larger in the left parietal lobe in the BMD I patients compared to BMD II patients (P < 0.05). In the temporal lobe, significant differences were observed in the spectrum distribution of beta and gamma frequency bands (P < 0.05).

**Conclusion:** The QEEG and entropy measure are simple and available tools to help detect cerebral cortex deficits and distinguish BMD I from BMD II.

Bipolar disorder is one of the severe and chronic disorders, involving mood changes, which may appear as elevated mood episodes (mania or hypomania) to low mood episodes (depression) (1). This disorder is often considered to have two ends one leading to bipolar I mood or classic disorder and the other end being sub-threshold mania or depression (2, 3). Bipolar II disorder is generally milder than bipolar I disorder, and according to DSM IV criteria, the number of criteria described for the both disorders is the same (1, 4, 5). The diagnostic criteria for these disorders are only based on the severity of symptoms; in the BMD II, performance will be slightly affected but in the BMD I the patients will have psychosis or less functions in various fields of life (4). However, both disorders have significant morbidity and mortality (6). Bipolar II disorder has more lifetime prevalence than bipolar I disorder (1, 4). Although the first accession in this disorder appears in late adolescence and early adulthood, this disorder is frequently seen in children and adolescents (7, 8). Despite the fact that the disorder appears in late adolescence and early adulthood with clear symptoms as episodic form, adolescents with bipolar disorder often have atypical and chronic symptoms without the episodes characterized by mania, hypomania and depression (7, 8). Therefore child or adolescent patients may experience elevated or low mood frequently during a day; and it is likely that they live with these symptoms without treatment for a few years and that the disorder may severely impact their educational and interpersonal performance and interfere with their personal and social development (8).

Accordingly, this disorder has a difficult diagnostic in childhood and adolescence. Therefore, under diagnosis and misdiagnosis is significant in this disorder (6, 9).

The QEEG as a common and reliable biomarker can be helpful in identification of neurophysiological differences in brain activity which are considered as main factors in occurrence of psychiatric disorders (10, 11, 12, 13, 14). The QEEG method is defined as “The mathematical processing of digitally recorded EEG in order to highlight specific waveform components, transform the EEG into a format or domain that elucidates relevant information, or associate numerical results” (15). Although many studies have examined the QEEG and brain function for several psychiatric disorders, there are a few studies on QEEG evaluation in patients with bipolar mood disorder (BMD), particularly in type I and type II, independently.

Oluboka and et al. evaluated absolute power and coherence differences with EEG signals features between patients with bipolar I disorder and schizophrenia (16). The results showed that right anterior hemisphere disorganization in BMD I patients is more than patients with schizophrenia. They also observed significant relationships between brain waves and presence of family history in the patients with BMD I. Bahrami et al. compared the fractal dimension of EEG signal in patients with BMD I through manic episode and normal subjects (17). They reported increases in brain complexity in BMD patients. Harmon-Jones et al. compared bipolar patients and normal individuals in relatively severe mental processes (18). They found higher left frontal activation in BMD patients.

In other studies, using EEG analysis and feature extraction from the signals, BMD diagnosis was done from other disorders such as schizophrenia and ADHD (19, 20, 21, 22). However, these studies were conducted without considering the type of bipolar disorder.

To the best of our knowledge, no studies have been conducted on patients with BMD I and BMD II and on their brain function, using QEEG analysis. While in some cases, the diagnosis of BMD I from BMD II is a serious challenge for psychiatrists, the aim of this study was to assess the differences in cortical function in these patients, using their EEG signal analysis.

## Material and Methods

*Participants*

In this study, 18 adolescents with BMD I (10 boys and 8 girls, 15.7 ± 1.50 years) and 20 adolescents with BMD II (10 boys and 10 girls, 16.1 ± 1.51 years), were diagnosed by two child and adolescent psychiatrists according to Diagnostic and Statistical Manual of Mental Disorders (DSM-IV-TR) criteria and were qualified for the study. Criteria leading to exclusion of patients from the study were as follows: Neurologic diseases such as seizure; head injuries; suspect to other mental disorders or having multiple disorders simultaneously; patients who took benzodiazepines or barbiturates; patients with a history of substance abuse; and reluctance of families to participate in the study.

*Experiment*

Electroencephalogram signals of the patients were recorded by SD-C24 QEEG device in the psychiatry and psychology research center in the Roozbeh hospital. According to 10-20 international system, the EEG signals were recorded by 21 electrodes in open and closed eyes at relax conditions and in a quiet room for about 10 minutes. The channels locations on the scalp were as follows: Fz, Cz, Pz, C3, T3, C4, T4, Fp1, Fp2, F3, F4, F7, F8, P3, P4, T5, T6, O1, O2, A1, A2. A1 and A2 channels average was used as reference. The signals are were filtered real time by a band pass FIR filter (a Butterworth filter with order 7) between 0.1 and 70 Hz and a notch filter at 50 Hz. Sampling frequency was set to 256 Hz. We had a pre-processing step to noise cancellation and artifacts reduction of the signals. Afterwards, 40 seconds segments were taken from the each pre-processed signals by an experienced neurologist using visual inspection (based on (23), this is an optimum length to extract the reliable spectral features from EEG signal). Thus, the final EEG segment had no artifacts such as EMG, EOG or head movement artifacts. In the next step, power spectrum density (PSD) was estimated from five frequency waves [i.e., delta (0.5-4 Hz), theta (4-8 Hz), alpha (8-12 Hz), beta (14-34 Hz) and gamma (34-44 Hz) bands], using periodogram Welch. Then, the features including power, mean, entropy (a measure of randomness in a signal), variance and skewness were extracted from PSD (23). Also, we were extracted the mean of the threshold spectrum (called component feature or PSD criteria) and the mean of the threshold spectrogram using short time Fourier transform or STFT (called STFT feature). Spectrogram is a visual time-variant spectral map of frequencies in the signal, and it is used extensively in the signal analysis in biomedical applications (18). The spectrogram of time series x(t) is defined as the magnitude of STFT. 


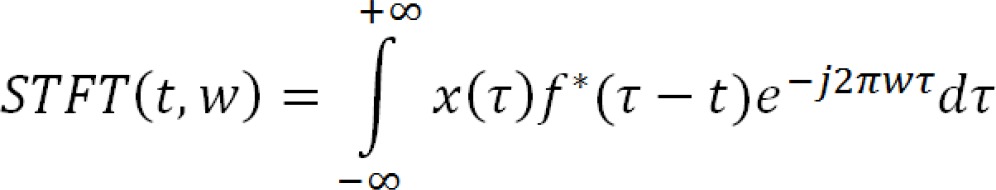


The STFT of signal x(t) is given by (24) where f(t) is a window function with short time duration and * indicates the complex conjugate. Based on the definition, the spectrogram is expressed as follows (18):


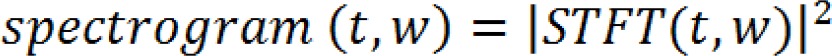


Based on the previously studies (18), this threshold was set to the maximum value of spectrum multiplied by 0.7. All of mentioned processes for EEG analysis were implemented by Matlab 8.2 (The Math works, Inc) in this study.

*Statistical Analysis*

According to data distribution, we used parametric and non-parametric tests to determine the statistically significant differences between patients with BMD I and BMD II. Hence, Shapiro-wilk test was used to assess our data distribution. Then, normal data was analyzed with independent t test and non-normal data were analyzed by Mann-Whitney test. We performed the statistical analysis using SPSS version 21. Significant difference level was set to at 0.05.

## Results

Table 1 summarizes the demographic information of the two groups. As shown in Table 1, no significant difference existed between the two groups in age and gender. Seven mentioned features were extracted from a preprocessed EEG segment for each subject.

**Figure 1:**
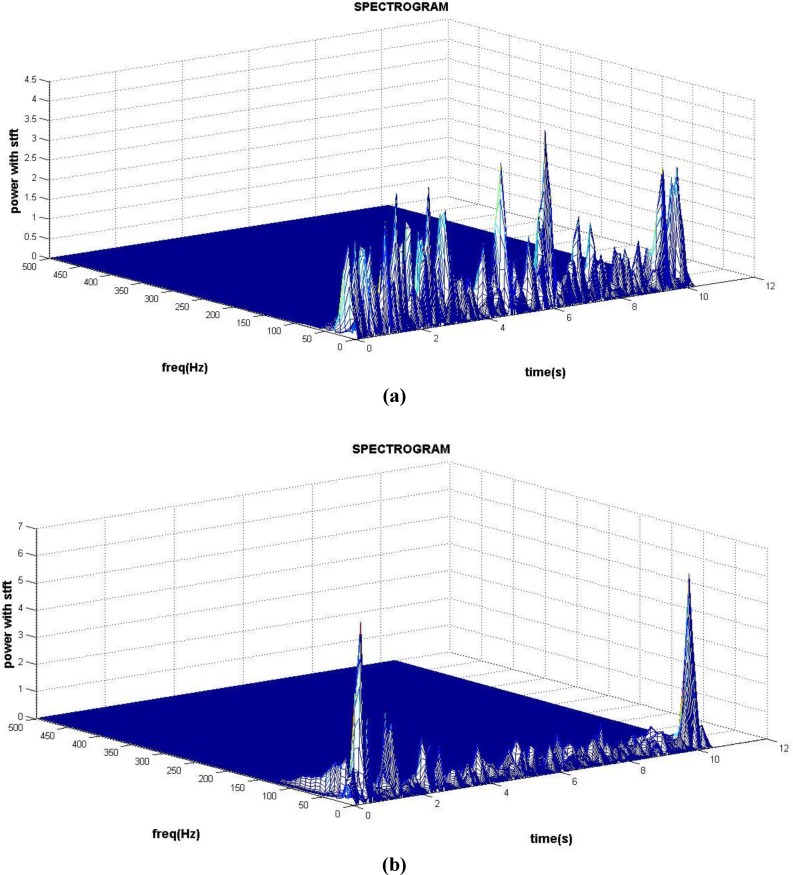
An Example of Spectrogram of P3 Electrode for a Patient with (a) BMD I and (b) BMD II

Due to stress and excessive head movement during recording, analysis was only done on the recorded signals in open eyes state. The BMD I had significant higher spectrogram criteria value at right prefrontal (Fp2) in beta band (P = 0.017), at right temporal (T6) in gamma band (P = 0.027), and at left occipital (O1) in delta band (P = 0.048). Examples of the spectrogram for BMD I and BMD II are shown for P3 electrode in Figure 1.

Although BMD I had higher spectral power in the most brain’s lobes, especially in parietal lobe, left parietal (P3) in delta band (P = 0.038) and mid-line frontal (Fz) in alpha band (P = 0.016) had only significant higher values versus BMD II. The average of EEG spectrum in the two groups showed significant differences with higher value at P3 electrode in delta band (P = 0.000) and F7 electrode in alpha band (P = 0.043) for BMD I.

Also, BMD I had also significant higher PSD criteria value at P3 (P = 0.014) and T4 (P = 0.000) electrodes in gamma band. The skewness of the frequency bands in the two groups was positive. However, BMD I had significant greater skewness at O2 electrode in alpha (P = 0.000) and gamma (P = 0.034) bands, T3 electrode in beta band (P = 0.024), T4 electrode in beta (P = 0.024) and gamma (P = 0.034) bands, and T5 electrode in beta band (P = 0.000). No significant difference was found between both the two groups in variance feature. 

**Table 1:** Demographic Information of Adolescents with Bipolar I and Bipolar II Disorders

**Parameters**	**BMD I (n = 18)**	**BMD II (n = 20)**	**P-value**	**t (df)**
Age	15.70±1.50	16.10±1.51	0.194	0.986 (37)
Gender (male, female)	10, 8	10, 10	0.391	-0.521 (37)
Handedness	Right (16), left (2)	Right (20)		

**Table 2:** Features with Significant Differences (P < 0.05) between Type I and Type II Bipolar Disorder based on Statistical Analysis

**Features**	**BMD I (Mean±S.D)**	**BMD II (Mean±S.D)**	**P-value**
FP1-gamma-entropy	0.581±0.298	0.010±0.000	0.000
FP2-beta-stft	0.416±0.185	0.651±0.183	0.017
F4-gamma-entropy	0.393±0.335	0.011±0.000	0.006
C3-alpha-entropy	0.764±0.043	0.831±0.103	0.043
C3-gamma-entropy	0.336±0.289	0.012±0.000	0.000
C4-gamma-entropy	0.645±0.207	0.483±0.225	0.027
P3-delta-mean	0.117±0.278	0.022±.069	0.000
P3-delta-power	0.115±0.265	0.021±0.009	0.038
P3-delta-entropy	0.399±0.184	0.363±0.068	0.048
P3-gamma-entropy	0.446±0.316	0.001±0.000	0.030
P3-gamma-component	0.976±0.009	0.984±0.012	0.014
O1-gamma-entropy	0.471±0.289	0.001±0.000	0.030
O1-delta-stft	0.712±0.234	0.496±0.286	0.048
O2-alpha-skewness	0.987±0.002	0.990±0.003	0.000
O2-gamma-skewness	0.985±0.007	0.980±0.005	0.034
O2-gamma-entropy	0.274±0.325	0.003±0.000	0.000
F7-alpha-mean	0.361±0.245	0.010±0.001	0.043
F7-gamma-entropy	0.383±0.325	0.002±0.000	0.014
T3-beta-skewness	0.895±0.043	0.936±0.052	0.024
T4-beta-skewness	0.895±0.039	0.933±0.048	0.024
T4-gamma-skewness	0.991±0.005	0.986±0.005	0.034
T4-gamma-component	0.966±0.011	0.977±0.010	0.000
T5-beta-skewness	0.904±0.042	0.938±0.051	0.000
T6-gamma-entropy	0.538±0.277	0.001±0.000	0.038
T6-gamma-stft	0.328±0.215	0.576±0.285	0.027
Fz-alpha-power	0.421±0.332	0.002±0.000	0.016

**Figure 2:**
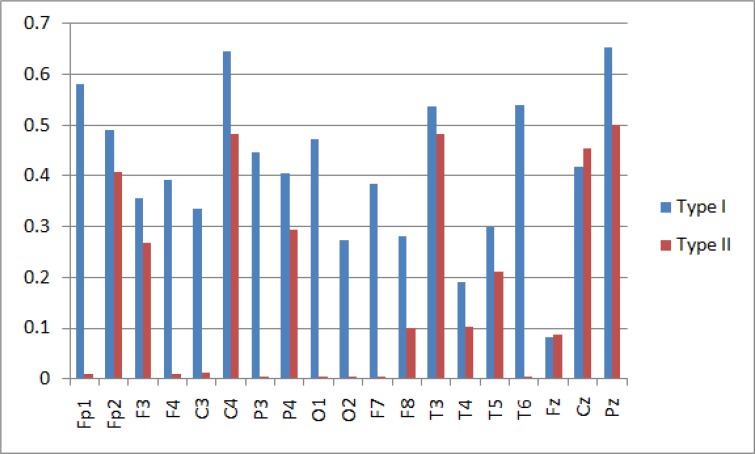
Spectral Entropy Values of Gamma Frequency Band in 19 Channels in Type I and Type II of BMD.

Table 2 demonstrates the mean and standard deviation of all significant features between patients with BMD I and BMD II.

The most number of significant differences between BMD I and BMD II groups have were found in spectral entropy, particularly in gamma frequency band. 

The BMD I had significantly higher spectral entropy values at FP1, F4, F7, C3, C4, P3, T6, O1 and O2 channels (P < 0.05) in gamma band significantly. Also, C3 electrode in alpha band (P = 0.043) and P3 electrode in delta band (P = 0.048) showed greater spectral entropy value in BMD I. Figure 2 displays the spectral entropy values of gamma band in all 19 electrodes for both the two groups.

## Discussion

According to our results, significant differences were found between BMD I and BMD II in the frontal lobe, central lobe, left parietal lobe, occipital lobe and temporal lobe. Generally, spectral entropy increases in BMD I for each lobe; this finding may indicate that gray matter in the BMD I has more deficits than BMD II. This observation is consistent with that of Ambrosi et al. and James et al. (25, 26). They found decreases in gray matter density of patients with BMD I and BMD II compared to normal subjects, respectively.

The frontal lobe is a progressive cognitive center, which integrates emotion and information to deal with inner and outer environment and extract episodic memory in the resting state (27). Based on our results, it seems that BMD I and BMD II patients have abnormalities in the prefrontal and frontal lobes, and this could explain the differences in executive function and emotional behavior between the subtypes of bipolar disorder. However, previously some studies suggested that the frontal and prefrontal regions are affected by mental disorders, particularly bipolar disorder (25, 28, 29, 30).

The temporal lobe which contains the hippocampus plays an important role in the formation of long-term memory; it is the advanced processing center for auditory, visual and semantic inputs (31). Temporal lobe damage can lead to behavioral changes and aggression, as well as lack of attention to the surrounding issues (31, 32). Liu et al. using resting state fMRI found that temporal gyrus is affected by BMD (29). This study revealed significant differences between BMD I and BMD II in the temporal lobe that either could indicate temporal lobe damage in the BMD or could explain some differences between these two types of disorders.

Parietal lobe functions include collection, and combine the sensory information from different parts of the body and process their relations (31). Parietal lobe damage can lead to sensory function and perception deficits (32). Also, left parietal lobe damage reduces the writing and math skills and sometimes speaking ability is also affected (27, 32). The results revealed significant differences between BMD I and BMD II in the left parietal lobe (P3 channel). However, entropy increases in this region for BMD I which may indicate more damage in patients with BMD I. However, Liang et al. suggested that right inferior parietal lobe is damaged with BMD which is inconsistent with our observation (27). The results of another study showed that patients with BMD have decreased cortical thickness within parietal lobe (28). 

Occipital lobe is the advanced processing center for the raw visual information (31). In this lobe, there are significant differences between both disorders including entropy and data distribution or skewness. Entropy increases in the BMD I on this region too. A similar observation was reported by Liang et al. (27) in the left occipital lobe for BMD.

**Limitation**

Small sample size was one limitation of this research. Thus, we reported preliminary findings, and to have more valid and conclusive results, replication of the study with larger population is recommended.

## Conclusion

In this paper, we presented a preliminary study about differences of electrical brain activity between BMD I and BMD II by EEG spectral analysis. In general, investigation of cerebral cortex function variations, using EEG signals, is a useful tool to identify the damaged brain regions in the bipolar disorder and compare the BMD I and BMD II. Although we used QEEG, we could only assess the cerebral cortex variations, while we are aware that deep brain damages lead to mental disorders. Yet, useful information is achieved from the brain damages in these disorders using QEEG analysis. Meanwhile, QEEG is always simply available and it is cost-effective for the patients.

In conclusion, The QEEG and entropy measure are simple and available tools to help detect cerebral cortex deficits and diagnose BMD I from BMD II.

## References

[1] Diagnostic and Statistical Manual of Mental Disorders (2000). Text Revision: DSM IV TR.

[2] Bauer M, Pfenning A (2005). Epidemiology of bipolar disorders. Epilepsia.

[3] Pini S, de Queiroz V, Pagnin D, Pezawas L, Angst J, Cassano GB ( 2005). Prevalence and burden of bipolar disorders in European countries.

[4] Sadock BJ, Sadock VA (2007). Kaplan & Sadock's Synopsis of Psychiatry.

[5] Hadjipavlou GH, Yatham L ( 2004). Bipolar II Disorder: an overview of recent developments. Canadian Journal of Psychiatry.

[6] Vieta E, Gastó C, Otero A, Nieto E, Vallejo J (1997). Differential features between bipolar I and bipolar II disorder. Comprehensive Psychiatry.

[7] Ghaemi SN, Ko JY, Goodwin FK (2002). "Cade's disease" and beyond: misdiagnosis, antidepressant use, and a proposed definition for bipolar spectrum disorder. Can J Psychiatry.

[8] Yatham L (2004). Diagnosis and management of patients with Bipolar II Disorder. Journal of Clinical Psychiatry.

[9] Angst J (2006). Do many patients with depression suffer from bipolar disorder?. Journal of Psychiatry.

[10] Chun J, Karam ZN, Marzinzik F, Kamali M, O'Donnell L, Tso IF, Manschreck TC, McInnis M, Deldin PJ (2013). Can P300 distinguish among schizophrenia, schizoaffective and bipolar I disorders? An ERP study of response inhibition. Schizophrenia research.

[11] Boutros NN (2011). Standard electroencephalography in clinical psychiatry: a practical handbook.

[12] Prichep LS, John ER (1992). QEEG profiles of psychiatric disorders. Brain topography.

[13] Shagass C, Roemer RA, Straumanis JJ (1982). (1982) Relationships between psychiatric diagnosis and some quantitative EEG variables. Archives of general psychiatry.

[14] Coutin-Churchman P, Añez Y, Uzcátegui M, Alvarez L, Vergara F, Mendez L, Fleitas R (2003). Quantitative spectral analysis of EEG in psychiatry revisited: drawing signs out of numbers in a clinical setting. Clinical Neurophysiology.

[15] Nuwer M (1997). Assessment of digital EEG, quantitative EEG, and EEG brain mapping: Report of the American Academy of Neurology and the American Clinical Neurophysiology Society*. Neurology.

[16] Oluboka OJ, Stewart SL, Sharma V, Mazmanian D, Persad E (2002). Preliminary assessment of intrahemispheric QEEG measures in bipolar mood disorders.

[17] Bahrami B, Seyedsajadi R, Babadi B, Nooroozian M (2005). Brain complexity increases in mania. Neuroreport.

[18] Harmon-Jones E, Abramson L Y, Nusslock, R, Sigelman JD, Urosevic S, Turonie LD (2008). Effect of bipolar disorder on left frontal cortical responses to goals differing in valence and task difficulty. Clinical Neurophysiology.

[19] Adeleh Dehghani Nazhvani, Reza Boostani, Somayeh Afrasiabi, Khadijeh Sadatnezhad (2013). Classification of ADHD and BMD patients using visual evoked potential. Clinical Neurology and Neurosurgery.

[20] Alimardani F, Boostani R, Azadehdel M, Ghanizadeh A, Rastegar K (2012). Presenting a new search strategy to select synchronization values for classifying bipolar mood disorders from schizophrenic patients. Elsevier, Engineering Applications of Artificial Intelligence.

[21] Sadatnezhad K, Boostani R, Ghanizadeh A, Classification of BMD (2011). ADHD patients using their EEG signals. Elsevier,Expert Systems with Applications.

[22] Venables NC, Bernat EM, Sponheim SR (2009). Genetic and disorder-specific aspects of resting state EEG abnormalities in Schizophrenia. Schizophr Bull.

[23] Gudmundsson S, Runarsson T, Sigurdsson S, Eiriksdottir G, Johnsen K (2007). Reliability of quantitative EEG features. Elsevier, Clinical Neurophysiology.

[24] Sheikhani A, Behnam H, Noroozian M, Mohammadi M, Mohammadi M (2009). Abnormalities of quantitative electroencephalography in children with Asperger disorder in various conditions. Research in Autism Spectrum Disorders.

[25] Ambrosi E, Rossi-Espagnet MC, Kotzalidis GD, Comparelli A, Del Casale A, Carducci F, Romano A, Manfredi G, Tatarelli R, Bozzao A, Girardi P (2013). Structural brain alterations in bipolar disorder II: a combined voxel-based morphometry (VBM) and diffusion tensor imaging (DTI) study. J Affect Disord.

[26] James A, Harley, J, Elisabeth Wells, Christopher M. A. Frampton, Peter R. Joyce (2011). Bipolar Disorder and the TCI: Higher Self-Transcendence in Bipolar Disorder Compared to Major Depression. Depress Res Treat.

[27] Liang M, Zhou Q, Yang K, Yang X, Fang J, Chen W, Huang Z (2013). Identify Changes of Brain Regional Homogeneity in Bipolar Disorder and Unipolar Depression Using Resting-State fMRI.. PLoS One.

[28] Lan MJ, Chhetry BT, Oquendo MA, Sublette ME, Sullivan G, Mann JJ, Parsey RV (2014). Cortical thickness differences between bipolar depression and major depressive disorder. Bipolar Disord.

[29] Liu CH, Li F, Li SF, Wang YJ, Tie CL, Wu HY, Zhou Z, Zhang D, Dong J, Yang Z, Wang CY (2012). Abnormal baseline brain activity in bipolar depression: a resting state functional magnetic resonance imaging study. Psychiatry Res.

[30] Xu K, Liu H, Li H, Tang Y, Womer F, Jiang X, Chen K, Zhou Y, Jiang W, Luo X, Fan G, Wang F (2014). Amplitude of low-frequency fluctuations in bipolar disorder: a resting state fMRI study. J Affect Disord.

[31] Frith CD, Frith U (1999). Interacting minds–a biological basis. Science.

[32] Lockwood KA, Alexopoulos GS, van Gorp WG (2002). Executive dysfunction in geriatric depression. Am J Psychiatry.

[33] Clementz B, Sponheim S, Iacono G, Beiser M (1994). Resting EEG in first - episode schizophrenia patients, bipolar psychosis patients, and their first- degree relatives. Psychophysiology.

[34] Koles J, Lind C, Flor-Henry P (1994). Spatial patterns in the background EEG underlying mental disease in man. Electroencephalogr. Clin. Neurophysiol.

[35] Schnack HG, Nieuwenhuis M, van Haren NE, Abramovic L, Scheewe TW, Brouwer RM, Hulshoff Pol HE, Kahn RS (2014). Can structural MRI aid in clinical classification? A machine learning study in two independent samples of patients with schizophrenia. Neuroimage.

